# Clinical Feasibility of Robotic-Assisted Endovascular Visceral Interventions

**DOI:** 10.1007/s00270-025-04340-z

**Published:** 2026-01-16

**Authors:** Julia Wagenpfeil, Patrick A. Kupczyk, Mathias Reinert, Jennifer Nadal, Carsten Meyer, Alexander Isaak, Claus C. Pieper, Julian A. Luetkens, Tatjana Dell, Daniel Kuetting

**Affiliations:** 1 Department of Diagnostic and Interventional Radiology, University Hospital of Bonn, Venusberg-Campus 1, 53127 Bonn, Germany; 2 Institute for Medical Biometry, Informatics and Epidemiology, University Hospital of Bonn, Venusberg-Campus 1, 53127 Bonn, Germany

**Keywords:** Robotics, Robotic-assisted vascular interventions, Radiation exposure, Women in interventional radiology, Pregnancy

## Abstract

**Purpose:**

Assessment of the clinical feasibility of robot-assisted endovascular visceral interventions to reduce physical strain caused by prolonged standing and enabling remote interventions.

**Material and Methods:**

Between 05/2024 and 09/2024, 45 patients were included in this prospective, single-center study. Patients scheduled for elective endovascular abdominal and pelvic interventions with superselective catheterization were assigned to manual (27 patients) or robotic-assisted treatment (18 patients). Radiation dose of the interventionalist, examination time (including preparation and follow-up), procedure duration and fluoroscopy time were compared between procedures using the CorPath GRX platform (Corindus, Waltham, MA) and conventional procedures. Technical success of robotic interventions was defined as achieving stable microcatheter positioning at the predefined target treatment point in the target vessel under robotic navigation, allowing execution of the planned therapy without conversion to manual navigation.

**Results:**

18 patients underwent robotic-assisted interventions (mean age 68 ± 12 years; 15 male), transarterial chemoembolization (TACE) (*n* = 9), ^99m^Tc-*MAA* simulation (MAA)/transarterial radioembolization (TARE) (*n* = 2) and prostatic artery embolization (PAE) (*n* = 7). 27 comparable procedures were performed manually (mean age 68 ± 10 years; 21 male): TACE (*n* = 13); MAA/TARE (*n* = 7); PAE (*n* = 7). 16/18 (88.9%; 95%-CI (Wilson) 67.3–96.7%) robotic-assisted procedures were technically successful, with manual conversion occurring in 2 patients (11.1%; 95%-CI (Wilson) 3.1–32.8%).

Neither median fluoroscopy time nor procedural dose, procedure duration or examination time differed between the robotic and conventional interventions [19 min (IQR 19.55) vs. 31 min (IQR 19.85); *p* = .053; 107.85 Gycm^2^ (IQR 164.03) vs. 128.00 Gycm^2^ (IQR 186.20); *p* = .286; 65 min (IQR 35.50) vs. 59 min (IQR 49.00); *p* = .711; 100 min (IQR 37.50) vs. 100 min (IQR 40.00); *p* = .853]. In comparison with conventional procedures, the operator’s dose was lower in robotic interventions [0.000 mSv (IQR 0.000) vs. 0.005 mSv (IQR 0.005); *p* < .001].

**Conclusion:**

Findings from this pilot case series indicate that robotic-assisted endovascular visceral interventions are feasible and demonstrate a high technical success rate, while simultaneously providing the interventionalist zero radiation exposure through remote operation from the control room.

**Graphical Abstract:**

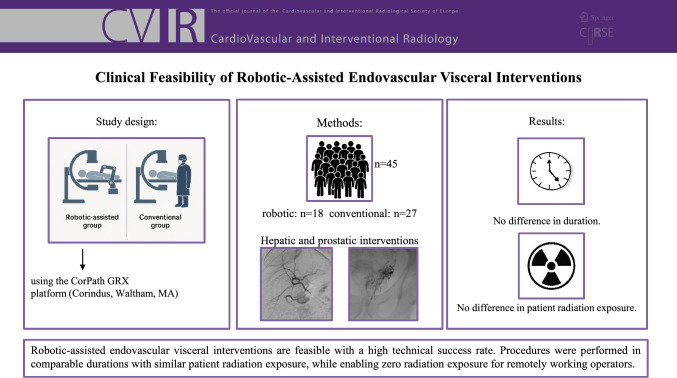

## Introduction

Robotic systems, integral to surgical procedures, also have the potential to drive groundbreaking innovation in interventional radiology (IR) [1–3].

Robotic systems for endovascular procedures enable precise superselective catheterization while rendering heavy lead aprons unnecessary and significantly reducing—or even eliminating—periprocedural radiation exposure for operators [2, 4]. These systems alleviate the physical demands of prolonged standing and allow interventions to be performed remotely.

These advantages particularly benefit the significantly under-represented women in interventional radiology, comprising only 2% of all interventional radiologists [5–8].

Despite the growing efforts to integrate robotic systems in endovascular procedures, experience remains mainly limited to neurovascular, cardiac and carotid interventions [9–15]. Although the CorPath GRX system (Corindus, Waltham, MA) has been successfully applied in these interventions, these non-visceral applications are still constrained by modest but non-negligible conversion rates to manual procedures, longer procedure times in some series, and small, highly selected cohorts, which supports a focused evaluation of its potential advantages in visceral embolization procedures such as TACE, PAE and TARE [16, 17]. While early feasibility studies and preclinical models conducted by our working group have demonstrated the potential of robotic guidance also in visceral interventions [2], no clinical data exist on the application of robotic-assisted techniques for superselective visceral interventions with this platform.

This study aimed to evaluate the feasibility and safety of robotic-assisted superselective endovascular interventions in the abdomen and pelvis in a selected subset of IR procedures.

## Materials and Methods

**Fig. 1:**
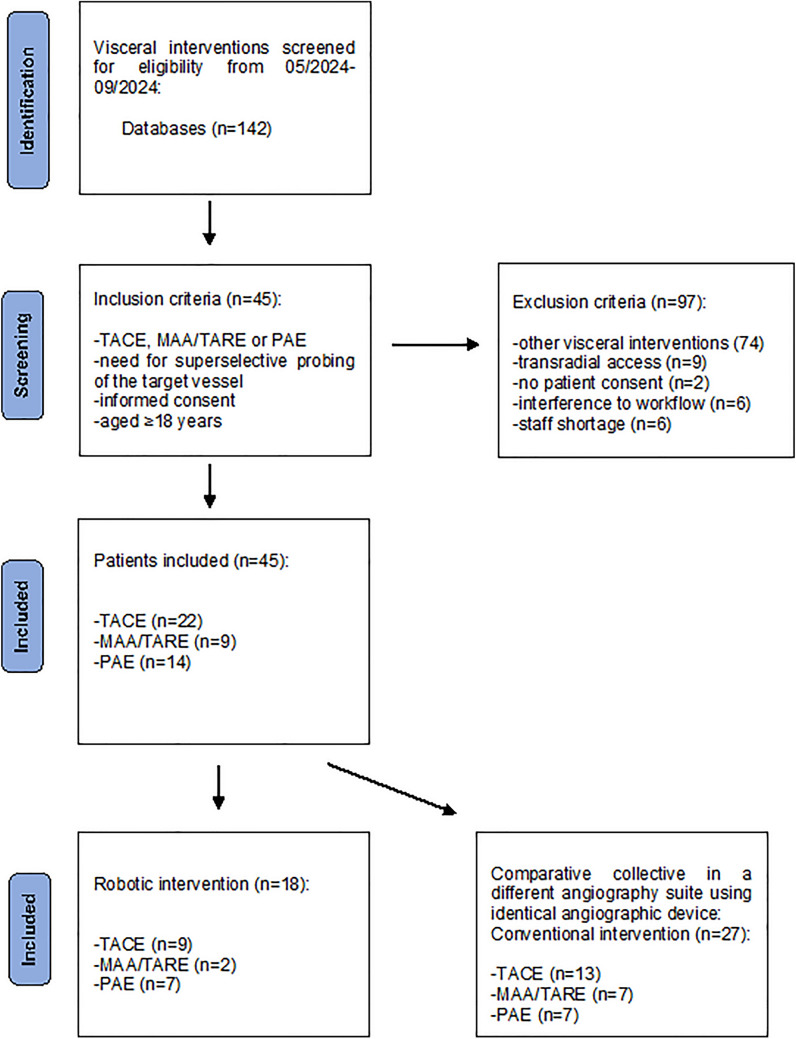
Study enrollment

This prospective study was approved by an institutional review board (University Hospital Bonn, application number 2024-30-BO). Participants gave written informed consent. Study enrollment is illustrated in Fig. 1.

### Patient Cohort

45 Patients scheduled for superselective interventional procedures between 05/2024 and 09/2024 were eligible for inclusion in the study. Inclusion criteria required patients to be aged ≥ 18 years and scheduled for one of the following interventions: prostatic artery embolization (PAE), transarterial chemoembolization (TACE), ^99m^Tc-*MAA* simulation (MAA), or transarterial radioembolization (TARE).

Exclusion criteria were interventions performed via transradial access, emergency cases and the lack of patient consent. 18 patients underwent robotic-assisted interventions; a cohort of 27 patients undergoing the same type of conventional procedures between 05/2024 and 09/2024 served as the control group (Table 1) and were selected based on the following criteria: Procedures were performed in a different angiography suite using an identical angiographic system without robotic assistance.

**Table 1:** Patient’s characteristics and radiation exposure

Variable	Robotic-assisted intervention	Conventional intervention	*p* Value
	Value (%)		
Number	18	27	
Sex			*p* = .721
Female	3 (16.7)	6 (22.2)	
Male	15 (83.3)	21 (77.8)	
Age^a^ (years)	68.2 ± 12	68.5 ± 9.8	*p* = .914
Clinical indication			
Hepatocellular carcinoma	8 (44.4)	12 (44.4)	
Cholangiocarcinoma	2 (11.1)	3 (11.1)	
Liver metastases	1 (0.6)	5 18.5)	
Benign prostatic hyperplasia	7 (38.9)	7 (25.9)	
Body Mass Index^a^	25.63 ± 4.3	27.32 ± 5.5	*p* = .301
Procedures			*p* = .512
TACE	9 (50)	13 (48.1)	
MAA/TARE	2 (11.1)	7 (25.9)	
Prostatic artery embolization	7 (38.9)	7 (25.9)	
Radiation exposure			
Operator dose^b^ (mSv)	0.000 [0.00]	0.005 [0.005]	*p* < .001
Examination dose^b^ (Gycm^2^)	107.85 [164.03]	128.00 [186.20]	*p* = .286
Fluoroscopy time^b^ (min)	19 [19.55]	31 [19.85]	*p* = .053
Procedure duration^b^ (min)	65 [35.50]	59 [49.00]	*p* = .711
Examination time^b^ (min)	100 [37.50]	100 [40.00]	*p* = .853

^**a**^Mean value ± standard deviation ^b^Median value [interquartile range]

Transarterial chemoembolization = TACE; technetium-labeled macroaggregated albumin simulation = MAA; transarterial radioembolization = TARE

Prior to inclusion in the study, eligible patients were scheduled consecutively and allocated 1:1 to one of the two angiography suites by simple randomization at the time of booking.

### Interventional Technique

**Fig. 2:**
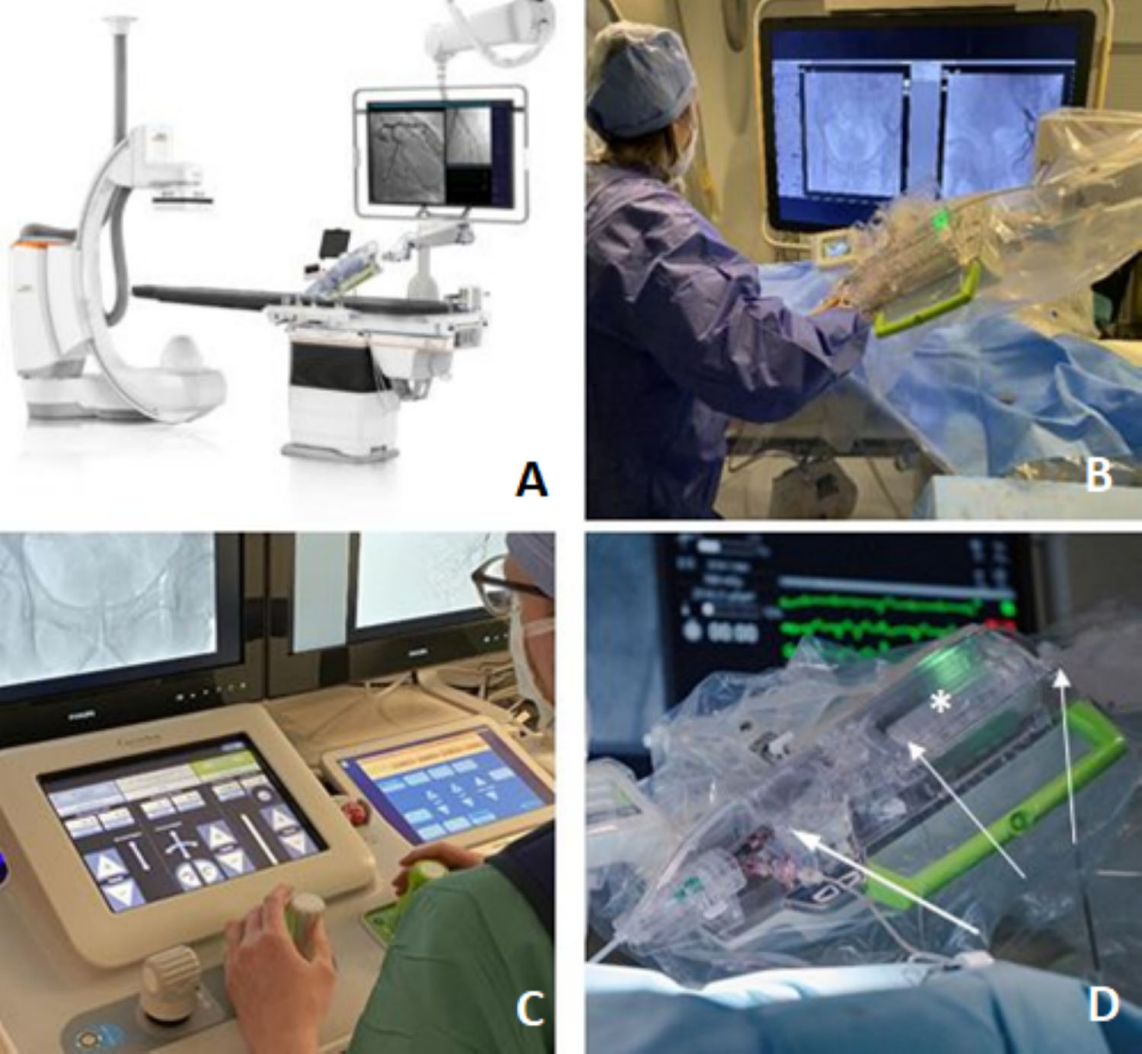
Setting for robotic interventions: Figure A (Image courtesy of Siemens Healthineers, used with permission) shows the general setting in the examination room with the bedside unit, Figure B the interventionalist at work in the examination room during catheter exchange. Figure C shows the interventional cockpit, which is operated from the control room. Figure D shows a close-up view of the bedside unit with the cassette (asterisk) and the microwire (arrows) inserted

**Fig. 3:**
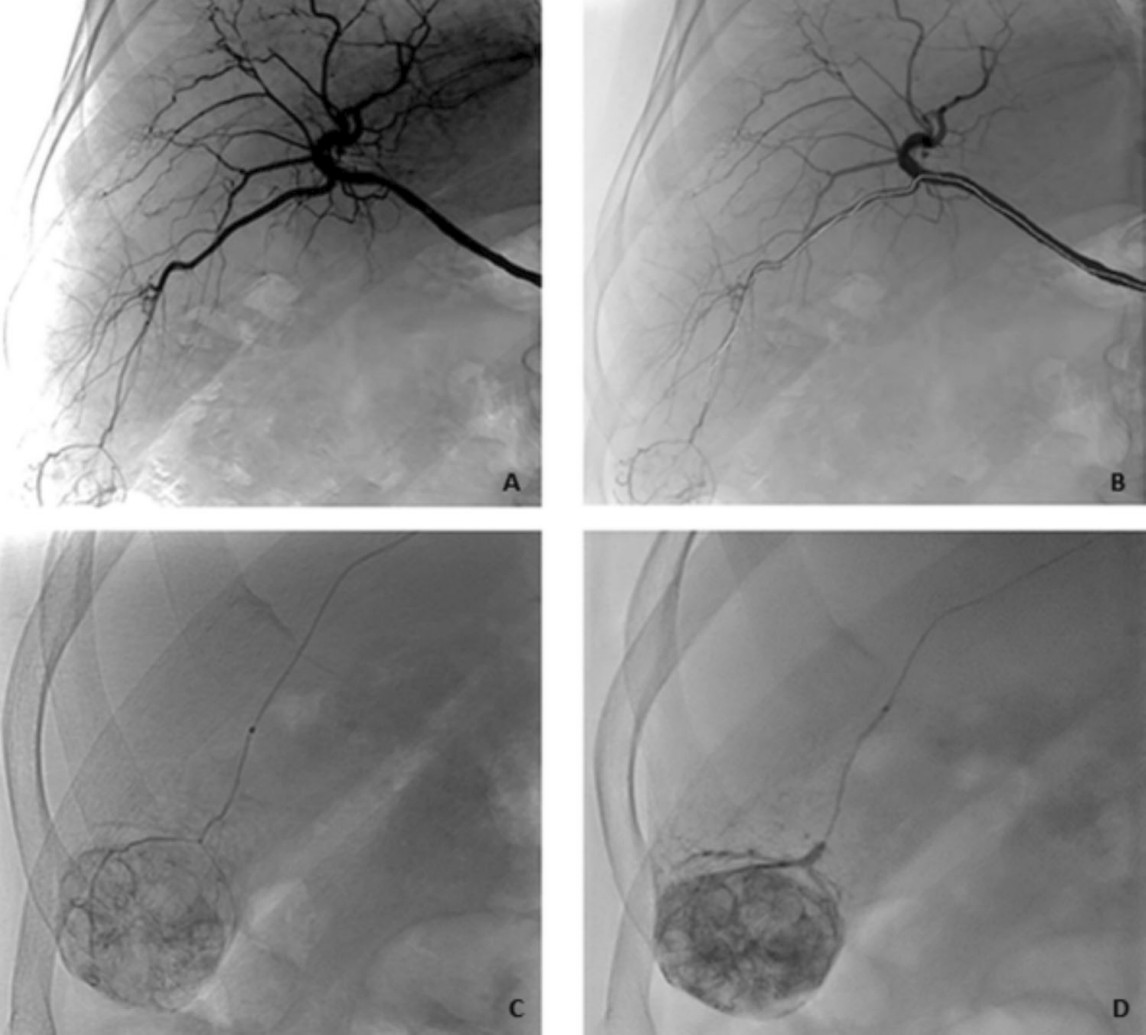
Chemoembolization in a 59-year-old patient with singular HCC lesion in segment VI. Figure **A** shows the overview angiography from the right hepatic artery. Figure **B** shows the measured section that was probed robotically using a microcatheter. Figure **C** shows the superselective microcatheter position in the segment VI supplying artery following robotic guided positioning. Image **D** shows lipiodol distribution following chemoembolization

**Fig. 4:**
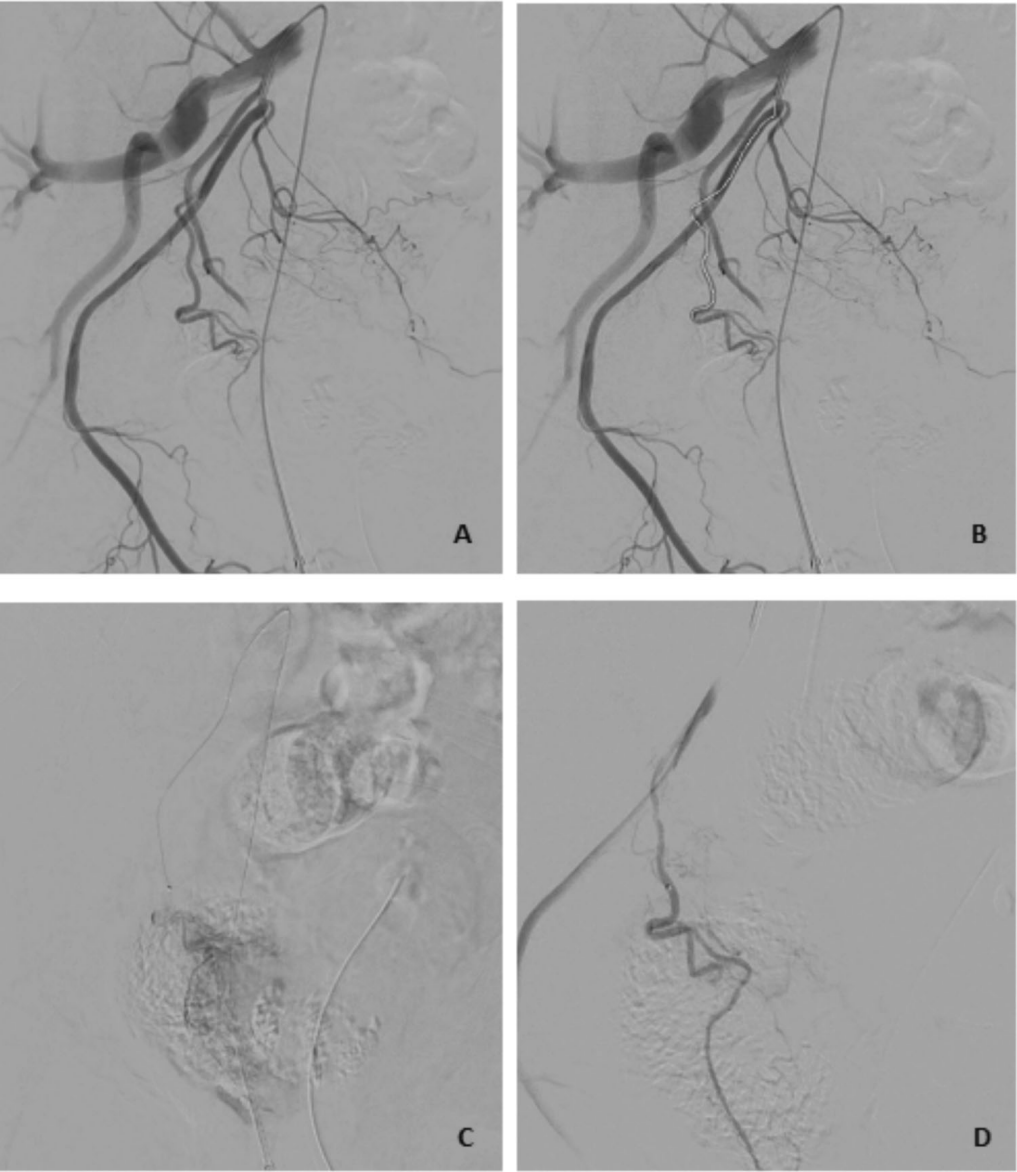
Prostate artery embolization of a 63-year-old patient. Figure **A** shows the overview angiography from the right-sided internal iliac artery (IIA) via the inserted macrocatheter. Figure **B** shows the route to be probed robotically using a microcatheter. Figure **C** shows the parenchymal blush of the prostate after successful cannulation of the prostatic artery with robotic guidance. Figure **D** shows the control after successful embolization (missing parenchymal blush)

Conventional angiographic procedures were performed by two interventionalists, one with 9 and the other with 6 years of experience in image-guided interventions. The same team prepared the robotic interventions and oversaw in-room operations. The robotic procedures themselves were carried out remotely by a third interventionalist with 3 years of experience in image-guided interventions and pregnant throughout the duration of this study. All robotic interventions were performed in the same angiography suite (Philips Allura Clarity, Amsterdam, The Netherlands) using the CorPath GRX platform (Corindus, Waltham, MA); main elements consist of a bedside robotic unit and a remote control console in the control room (Fig. 2), as described previously in detail [2, 12, 18]. All three interventionalists had only limited experience with the robotic platform. Prior to initiation of robotic interventions, manual creation of an inguinal access, placement of vascular sheath and a 4F-or 5F-macrocatheter into the proximal target vessel (Cobra C2 or SIM1, Cordis, Brussels, Belgium) was performed. For TACE and MAA, the proximal target vessel was typically the common hepatic artery, for PAE the internal iliac artery (IIA). After coaxial insertion of a microcatheter (Renegade or Direxion (Bern shape)), Boston Scientific, Marlborough, MA) and a 0.014-inch guidewire (Syncro, Stryker, Kalamazoo, MI), the robotic system was connected, and the distal target vessel was catheterized superselectively using the robotic unit. Depending on the procedure, the right and/or left prostate artery or the tumor-feeding hepatic segmental artery were catheterized (Fig. 3, 4). The particles for MAA/TARE, TACE and PAE were administered manually. For ipsilateral PAE, the robotic system was temporarily disconnected to manually catheterize the ipsilateral IIA via Waltman Loop maneuver [19]. Robot preparation and sterile draping were performed simultaneously to manual creation of femoral vascular access and conventional catheterization. Conventional interventions were performed accordingly via manual superselective microcatheter cannulation of the distal target vessel using the same materials and were performed in a different angiography suite equipped with an identical angiographic system (Philips Allura Clarity, Amsterdam, The Netherlands).

### Data Acquisition and Definitions

Clinical and periprocedural data were collected and compared as follows (Table 1):

Radiation exposure: Live dosimetry (ALADOX, Automess GmbH, Ladenburg, Germany) was used to monitor the dose of the interventionalist operating the robot from the control console who did not wear protective lead gear throughout the procedures. Likewise, for the control group, live dosimetry was performed for the interventionalist performing conventional angiography; dosimeters were positioned beneath protective lead gear at chest level with a lead equivalent of 0.50mmPb; further radiation protection such as a lead glass pane (0.50 mmPb) and table-mounted lead shield (0.50 mmPb) was used. Mean applied procedural dose was analyzed and compared.

Fluoroscopy times, mean examination times (including preparation and follow-up) and procedure duration (the procedure as such) were measured in minutes and correlated. A 30-day clinical follow-up was conducted post-intervention.

Technical success of robotic interventions was defined as achieving stable microcatheter positioning at the predefined target treatment point in the target vessel under robotic navigation, allowing execution of the planned therapy without conversion to manual navigation.

### Statistical Analysis

Statistical analyses were performed employing SPSS, V29.0 (IBM,NY). Descriptive statistics were done for patient characteristics and procedural parameters. The gender distribution and frequency of the therapies were given as a percentage. Normal distribution was assessed using Q-Q plots for all parameters. Continuous normally distributed variables are given as mean and standard deviation, continuous non-normally distributed variables as median and interquartile range (IQR). Age and Body Mass Index were compared between the two groups using the T test for independent samples. Gender distribution and proportion of procedures were compared with the Chi-square test. The operator dose, examination dose, fluoroscopy time, procedure duration and examination time were compared between robotic and conventional interventions using the Mann–Whitney U-test. *P* values of < 0.05 were considered statistically significant. The effect sizes were determined. Statistical tests were two-sided and considered exploratory. No formal adjustment for multiple comparisons was performed; therefore, *p* values should be interpreted descriptively, particularly in subgroup analyses.

## Results

### Patient Cohort

In total, 18 patients (15 male; mean age 68.2 ± 12 years) underwent robotically assisted minimally invasive procedures. 9/18 (50%) patients received TACE, 2/18 (11.1%) patients received MAA for TARE planning and 7/18 (38.9%) patients underwent PAE via superselective robotic catheterization.

Twenty-seven patients (21 male; mean age 68.5 ± 9.8 years) were included in the control group and underwent conventional intervention. 13/27 (48.1%) patients received TACE, 7/27 (25.9%) patients received MAA or TARE, and 7/27 (25.9%) underwent PAE via superselective robotic catheterization (Table 1).

### Interventional Treatment

After manual creation of an inguinal access and placement of a 4F- or 5F-macrocatheter into the proximal target vessel the microcatheter was navigated robotically to the distal target vessels. During this process, control angiograms showed no evidence of dissection or perforation. Microcatheter position remained stable during all robotic-assisted therapies without any dislocation being noted. In 2/18 cases (11.1%), a conversion to manual intervention was necessary. Overall, 16/18 procedures (88.9%) were technically successful, and no complications occurred. There were also no complications performing conventional interventions, and the technical success rate was 27/27 (100%).

Total examination time (including preparation time) and procedure duration of the robotic interventions did not differ from the conventionally performed interventions and showed small effect sizes [100 min (IQR 37.50) vs. 100 min (IQR 40.00); *p* = 0.853; *r* = 0.0277; 65 min (IQR 35.50) vs. 59 min (IQR 49.00); *p* = 0.711, *r* = 0.055].

### Radiation Exposure

Median applied procedural dose showed no significant differences between robotic and conventional interventions with a small effect size [107.85 Gycm^2^ (IQR 164.03) vs. 128.00 Gycm^2^ (IQR 186.20); *p* = 0.286; *r* = 0.159].

Median operator’s dose sitting in the control room in robotic interventions was lower than the median operator’s dose for the conventional procedures in the angiography suite [0.000 mSv (IQR 0.000) vs. 0.005 mSv (IQR 0.005); *p* < 0.001]. The effect size r was 0.869, indicating a large effect. Median fluoroscopy time was not significantly different between robotic and conventional interventions [19 min (IQR 19.55) vs. 31 min (IQR 19.85); *p* = 0.053]. The effect size r was 0.288, indicating a medium-sized effect. There were no complications during the clinical follow-up period of 30 days or subsequent complaints.

### Hepatic Interventions

**Fig. 5:**
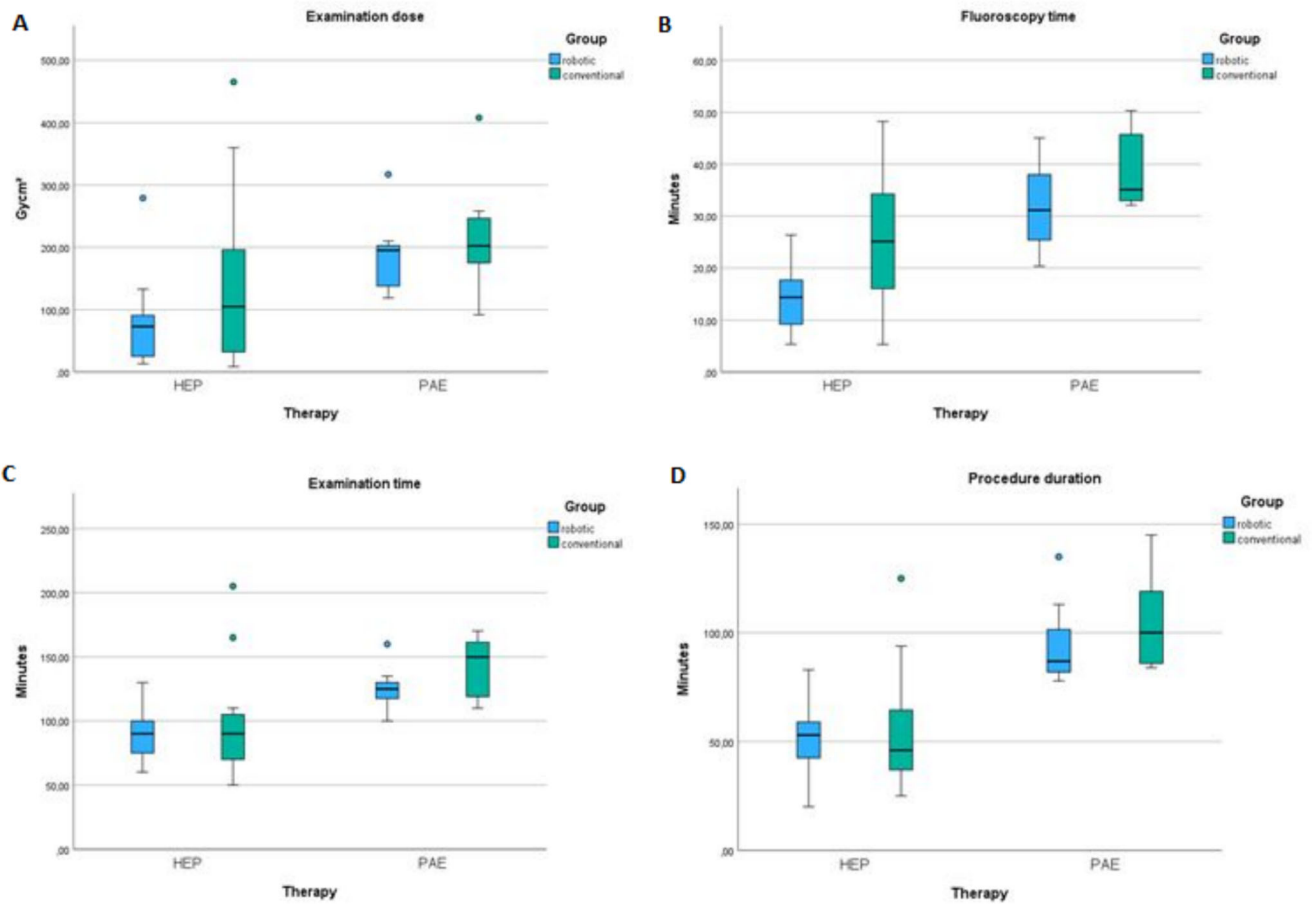
Subgroup analysis for hepatic and prostatic intervention in robotic and conventional treated patients regarding examination dose (**A**), fluoroscopy time (**B**), total examination time (**C**) and procedure duration (**D**)

In subgroup analysis, the median examination dose, median total examination time, as well as procedure duration in 11 patients who received robot-guided TACE, MAA or TARE and in 20 patients who received conventional TACE, MAA or TARE showed no significant differences (Fig. 5a, c, d). Median operator’s dose sitting in the control room in robotic hepatic interventions was lower than the median operator’s dose for the conventional interventions in the angiography suite; median fluoroscopy time was lower for robotic hepatic interventions than in conventional interventions (Fig. 5b).

In 1/11 (9.1%) hepatic interventions, a cassette malfunction required conversion to conventional technique. The wire could be removed from the cassette; the macro- and microcatheter disconnected. The conventionally performed angiography could be successfully completed without complications. 10/11 (90.9%) hepatic procedures could be performed technically successful. All robotic-assisted MAA/TARE interventions were technically successful (100%).

### PAE

In subgroup analysis, the median examination dose, the median fluoroscopy time, total examination time and procedure duration in 7 patients who received robot-guided PAE and in 7 patients who received conventional PAE showed no significant differences (Fig. 5a–d). Median operator’s dose sitting in the control room in robotic PAE was lower than the median operator’s dose for the conventional PAE in the angiography suite. In 1/7 PAE procedures (14.3%; 1 out of 14 prostatic arteries), robotic advancement of the microcatheter for ipsilateral embolization was impeded by excessive friction with the macrocatheter, most likely due to a steep catheter curve (Waltman loop maneuver) [19], following successful contralateral PAE. After disengaging the robotic system, manual advancement of the microcatheter was successfully achieved without the need to reposition the microcatheter. 6/7 (85.5%; 13/14 prostatic arteries) PAE procedures were technically successful.

## Discussion

In the present study, the technical feasibility of robotic-assisted visceral interventions, including PAE, TACE, and MAA/TARE, was evaluated and showed a high overall success rate (88.9%). No differences in procedural duration or total radiation dose were noted compared to conventional interventions. The interventionalist performing the robotic procedures did so remotely without being exposed to any periprocedural radiation. Similar to surgical procedures, where robotic assistance was successfully implemented more than a decade ago, the use of robotics in catheter-based visceral interventions offers the prospect of improved precision, enhanced accuracy, and reduction of procedural errors [20, 21]. Previous studies have proven the feasibility of robotic-assisted visceral superselective interventions in animal models [2, 22, 23]. The introduction of robotic systems in catheter-based IR has the potential to offer significant advancements in operator safety and procedural efficiency, as already demonstrated with other robotic platforms [18, 20, 24, 25]. A key benefit is the complete elimination of radiation exposure for the interventionalist, as confirmed by a recorded dose of 0.000 mSv for the interventionalist performing during all robotic interventions. Eliminating the need for operators to wear heavy lead aprons during complex visceral procedures involving challenging vascular anatomy can reduce operator fatigue and may contribute to improved outcomes [2, 26, 27]. In addition to similar procedure durations, patient radiation doses during robotic interventions remained comparable to those of conventional interventions, consistent with previously reported findings for robotic-assisted coronary interventions [27–29]. Contrary to previous reports from studies investigating robotic-assisted interventions in non-visceral settings, no differences were found in the mean examination time as well as procedure duration [27–29]. This may be attributed to the parallel workflow in our study, where the time-consuming preparation of the robotic arm was conducted simultaneously to groin access and manual catheterization of the proximal target vessel with the macrocatheter. Despite the interventionalist’s limited experience with the robotic platform at the study outset, remote microcatheter guidance proved intuitive, as evidenced by the comparable fluoroscopy time [30]. While not formally assessed, staff radiation exposure is likely slightly reduced during robotic interventions due to increased distance from the patient. The manual conversion rate in our study was 11.1%, a value inherently influenced by procedural complexity and vascular anatomy, both of which pose challenges for robotic intervention. Despite the demanding cannulation conditions, particularly in ipsilateral prostate procedures, the rate of manual assistance remained at the lower end compared to similar studies [31–33]. Despite significant improvements in radiation protection in recent years, daily radiation exposure still poses significant risks, especially female physicians in childbearing age [33]. The physical demands of prolonged procedures, including the burden of heavy protective gear, further compound these challenges. While 0.5 mmPb lead aprons reduce scattered radiation, many pregnant interventionalists avoid exposure entirely, aligning with practices in other surgical specialties [3, 34–38]. Robotic-assisted interventions offer a solution, addressing safety while supporting career continuity and more inclusive work environment, ultimately supporting greater gender diversity in IR [5–8].

Despite its innovative potential, this technology still presents notable limitations. System setup and instrument replacement demand specialized training and increase procedural effort and costs. Beyond the high acquisition cost of the robotic system itself, the substantial expense of the required cassettes must also be considered, as these are currently not reimbursed by most healthcare system.

The CorPath GRX platform has technical limitations relative to other robotic systems. In contrast to the Magellan system (Hansen Medical, Mountain View, California, USA), the shape and angulation of the catheter cannot be adjusted remotely, and contrast injection requires manual syringe reconnection or combination with a contrast agent injector. The Magellan system, however, is not designed for superselective catheterization of distal target vessels, whereas the system used in our study enables such superselective access. Further limitations include the restricted compatibility of the employed robotic system with catheters and guidewires (e.g., necessity of a 100-cm-long macrocatheter and a > 200 cm microwire).

Furthermore, the absence of tactile feedback gives rise to worries regarding inadvertent harm to blood vessels, though complications were neither noted in prior animal studies nor in the current study [2, 18]. Only selected interventions were tested in our study. Complex procedures, such as the recanalization of visceral arteries—which require extensive manipulation—or the embolization of acute visceral hemorrhages—which demand speed—may present challenges and warrant further investigation. In our study, the presence of an additional radiologist in the examination room was primarily dictated by the study protocol including a pregnant interventionalist. Otherwise, the interventionalist operating the robot could have also completed the two cases requiring conversion to a manual approach. The current necessity of an experienced interventionalist on-site challenges the feasibility of fully remote-controlled interventions, given the limitations of existing technology [28].

Currently, the employed robotic device is not commercially available in its present form; however, further advancements in robotic angiography are anticipated to enhance usability and procedural efficiency. This study is a non-randomized study, limited by the small size of the reported study sample.

In addition, no intra-individual comparison was performed in our study, so that patient selection may have had an influence on the results.

Findings from this pilot case series demonstrate that robotic-assisted endovascular visceral interventions are feasible with a high technical success rate. Procedures were performed in comparable durations and with similar patient radiation exposure, while enabling zero radiation exposure for remotely operating interventionalists—an advantage highlighted in our study by the participation of a pregnant operator.

## Abbreviations

IR: Interventional radiology
TACE: Transarterial chemoembolization
MAA: Technetium-labeled macroaggregated albumin simulation
TARE: Transarterial radioembolization
PAE: Prostatic artery embolization
IIA: Internal iliac artery
